# Rabbit-specific computational modelling of ventricular cell electrophysiology: Using populations of models to explore variability in the response to ischemia

**DOI:** 10.1016/j.pbiomolbio.2016.06.003

**Published:** 2016-07

**Authors:** Philip Gemmell, Kevin Burrage, Blanca Rodríguez, T. Alexander Quinn

**Affiliations:** a Department of Computer Science, University of Oxford, Oxford, UK; b School of Mathematical Sciences and ARC Centre of Excellence, ACEMS, Queensland University of Technology, Brisbane, Australia; c Department of Physiology and Biophysics, Dalhousie University, 5850 College St, Lab 3F, Halifax, NS B3H 4R2, Canada; d School of Biomedical Engineering, Dalhousie University, 5850 College St, Lab 3F, Halifax, NS B3H 4R2, Canada

**Keywords:** Cardiac cell electrophysiology, Computational modelling, Ischemia, Populations of models, Rabbit, Variability

## Abstract

Computational modelling, combined with experimental investigations, is a powerful method for investigating complex cardiac electrophysiological behaviour. The use of rabbit-specific models, due to the similarities of cardiac electrophysiology in this species with human, is especially prevalent. In this paper, we first briefly review rabbit-specific computational modelling of ventricular cell electrophysiology, multi-cellular simulations including cellular heterogeneity, and acute ischemia. This mini-review is followed by an original computational investigation of variability in the electrophysiological response of two experimentally-calibrated populations of rabbit-specific ventricular myocyte action potential models to acute ischemia. We performed a systematic exploration of the response of the model populations to varying degrees of ischemia and individual ischemic parameters, to investigate their individual and combined effects on action potential duration and refractoriness. This revealed complex interactions between model population variability and ischemic factors, which combined to enhance variability during ischemia. This represents an important step towards an improved understanding of the role that physiological variability may play in electrophysiological alterations during acute ischemia.

## Abbreviations

2D: two-dimensional
AP: action potential
APD: action potential duration
APD_90_: APD at 90% repolarisation
[Ca^2+^]_i_: intracellular Ca^2+^ concentration
DAD: delayed after-depolarisation
d*V*_m_/dt_max_: maximum rate of *V*_m_ change
EAD: early after-depolarisation
ECG: electrocardiogram
ERP: effective refractory period
*f*: current conductance scaling factor
*g*: variable current conductance
*h*,*j*: *I*_Na_ inactivation gates
*I*_Ca,L_: L-type Ca^2+^ current
*I*_K1_: inward rectifier K^+^ current
*I*_K,ATP_: ATP-inactivated K^+^ current
*I*_Kr_: rapid delayed rectifier K^+^ current
*I*_Ks_: slow delayed rectifier K^+^ current
*I*_Na_: fast Na^+^ current
*I*_NaK_: Na^+^-K^+^ pump current
*I*_NCX_: Na^+^-Ca^2+^ exchanger current
*I*_stim_: stimulus current
*I*_to_: transient outward potassium current
[K^+^]_o_: extracellular potassium concentration
SR: sarcoplasmic reticulum
*V*_m_: membrane potential
*V*_max_: maximum *V*_m_
*V*_rest_: resting *V*_m_

## 1 Introduction

### 1.1 Rabbit-specific computational modelling of cardiac electrophysiology

Computational modelling is an increasingly powerful tool, especially when combined with experimental investigations, for the understanding of complex cardiac electrophysiological behaviour (Carusi et al., 2012, Fink et al., 2011, Noble and Rudy, 2001, Quinn and Kohl, 2013, Roberts et al., 2012, Trayanova, 2011). As with any model (whether it be computational, experimental, or conceptual), computational models of cardiac electrophysiology represent current collective understanding and are designed for specific applications (Bers and Grandi, 2011, Clancy et al., 2016, Noble, 2011, Quinn and Kohl, 2011, Trayanova et al., 2006, Vigmond and Stuyvers, 2016, Winslow et al., 2012). While models exist for a variety of species, rabbit-specific models are a prevalent small animal model, as rabbit cardiac electrophysiology is generally more similar to human than that of small rodents (Bers, 2002, Nattel et al., 2008, Nerbonne, 2000). They are highly relevant for understanding human pathophysiology (Janse et al., 1998, Lawrence et al., 2008), as, for instance, responses of the rabbit heart to ischemia and to pharmacological interventions are also more similar to human than other small animal models (Harken et al., 1981) and the ratio of rabbit heart size to excitation wavelength, which dramatically affects arrhythmogenic wave patterns, is closer to human than even dog or pig (Panfilov, 2006).

In this paper, we first review existing rabbit-specific computational models of cardiac cell electrophysiology and their use in multi-cellular simulations exploring the influence of physiological and pathological cellular heterogeneity on electrical activity, to establish their utility and the current state-of-the-art. This is followed by an original investigation presenting a new methodology using experimentally-calibrated populations of rabbit-specific computational models for the study of variability in the electrophysiological response to acute ischemia, which represents an innovative tool for future studies of the contribution of physiological variability to arrhythmic risk.

### 1.2 Rabbit-specific modelling of ventricular cell electrophysiology

Numerous biophysically-detailed action potential (AP) models for specific rabbit heart cell types have been generated. Based on the abundance of available experimental data, the earliest and most prevalent are sinoatrial node cell models, which have been vital for the integration and interpretation of results, helping explain key mechanisms underlying the beat-to-beat regulation of cardiac pacemaker function (as reviewed by others (Ravagli et al., 2016, Wilders, 2007)). Rabbit-specific models of atrioventricular node (Inada et al., 2009, Liu et al., 1993), Purkinje (Corrias et al., 2011, Inada et al., 2009) and atrial (Hilgemann and Noble, 1987, Lindblad et al., 1996) cell electrophysiology also exist, but their use for the study of physiological and pathological function has been limited. The first rabbit-specific ventricular myocyte model was developed by Puglisi and Bers (2001), and has been revised as new knowledge regarding cellular function has become available (Mahajan et al., 2008b, Morotti et al., 2012, Shannon et al., 2004, Shannon et al., 2012).

#### 1.2.1 Puglisi-Bers model

The Puglisi-Bers rabbit ventricular cell model was developed as an interactive computer program (LabHEART), to provide an easily accessible tool for student learning and for researchers to explore experimentally-testable hypotheses (Puglisi and Bers, 2001). The model was a rabbit-specific modification of the Luo-Rudy guinea pig model (Luo and Rudy, 1991, Luo and Rudy, 1994, Zeng et al., 1995), the most commonly used ventricular cell model at the time. Changes to the model included: (i) introduction of transient outward K^+^ (*I*_to_) and Ca^2+^-activated chloride currents; (ii) adjustment of T-type Ca^2+^ and rapid delayed rectifier K^+^ (*I*_Kr_) currents’ kinetics; (iii) and rescaling of fast Na^+^ (*I*_Na_), inward rectifier K^+^ (*I*_K1_), plateau K^+^, and Na^+^-Ca^2+^ exchanger (*I*_NCX_) currents’ conductance to reproduce the electrophysiological and Ca^2+^ transport characteristics of rabbit ventricular myocytes.

Control simulations demonstrated that the model reproduced normal rabbit ventricular myocyte currents and AP and Ca^2+^ transient morphology. The model was then used to simulate heart failure by reducing *I*_to_, *I*_K1_, and sarcoplasmic reticulum (SR) Ca^2+^-ATPase activity and increasing *I*_NCX_. Simulations helped further validate the model, while defining the cellular basis of heart failure-induced changes in AP and Ca^2+^ transient morphology and the propensity for triggered arrhythmias. Simulations reproduced the increase in AP duration (APD; especially at longer cycle lengths) and the reduction in Ca^2+^ transient amplitude that are observed experimentally. They demonstrated that the increase in *I*_NCX_ or decrease in *I*_K1_ with heart failure equally lower the intracellular Ca^2+^ concentration ([Ca^2+^]_i_) threshold for Ca^2+^-triggered excitation, and in combination have a nearly additive effect.

The fact that the model was able to reproduce electrophysiological activity of both normal and heart failure rabbit ventricular cells, and be used to investigate the contribution of different factors to arrhythmogenesis, represented a major step forward in the use of computational modelling to study physiological and pathological function with rabbit experimental models. It should be noted, however, that not all aspects of the Luo-Rudy model were updated based on rabbit data (most notably, ion fluxes involved in intracellular Ca^2+^ handling were generally left unmodified, as was the Na^+^-K^+^ pump current, *I*_NaK_). This lack of species-specificity highlights the fact that model ‘re-use’ is common, so that even ‘species-specific’ models are based on experimental data acquired from a host of different species (under different experimental conditions, in various preparations), and as a result some outputs may differ from actual function (Niederer et al., 2009).

#### 1.2.2 Shannon model

The first modification of the Puglisi-Bers model came from Shannon et al., which updated the balance of Ca^2+^ removal mechanisms (*i.e.*, SR Ca^2+^-ATPase *vs*. Na^+^-Ca^2+^ exchange activity) to match data from rabbit ventricular cells and to reproduce the nonlinear dependence of gain and fractional SR Ca^2+^ release on SR Ca^2+^ load (Shannon et al., 2004, Shannon et al., 2012). Specifically, the model was modified to include: (i) a sub-sarcolemmal compartment (in addition to the existing junctional and bulk cytosolic compartments - the first computational model to do this); (ii) updated cytosolic Ca^2+^ buffering parameters; (iii) a reversible SR Ca^2+^ pump; (iv) a scheme for Na^+^-Ca^2+^ exchange transport that is dependent on intracellular Na^+^ concentration ([Na^+^]_i_) and allosterically regulated by [Ca^2+^]_i_; and (v) a practical model of SR Ca^2+^ release including both inactivation/adaptation and SR Ca^2+^-load dependence.

Most significantly, the last feature, that binding of Ca^2+^ to ryanodine receptors on their SR luminal site increased the affinity of the cytosolic activation site for Ca^2+^, while simultaneously decreasing the affinity of the cytosolic inactivation site, allowed the model to reproduce experimentally-observed relationships between SR Ca^2+^ load and characteristics of SR Ca^2+^ release. The model was used in subsequent simulations to generate the important hypothesis that ryanodine receptor regulation by luminal Ca^2+^ adjusts steady-state SR Ca^2+^ levels in response to altered ryanodine receptor Ca^2+^ sensitivity (Shannon et al., 2005). The model, however, suffered from its reliance (in part) on cytosolic Ca^2+^-dependent inactivation for termination of SR Ca^2+^ release, and failed to exhibit Ca^2+^ transient alternans at rapid stimulation rates, important for arrhythmogenesis.

#### 1.2.3 Mahajan model

To address the above deficiencies, the Shannon model was revised by Mahajan et al. to include changes in *I*_Ca,L_, intracellular Ca^2+^ cycling, *I*_NCX_, and channel distributions to better replicate rabbit AP and Ca^2+^-handling dynamics at rapid stimulation rates (Mahajan et al., 2008b). The most significant of these changes was the inclusion of a Markovian model of *I*_Ca,L_, which reproduced Ca^2+^- and voltage-dependent kinetics measured in isolated rabbit ventricular myocytes, in combination with a previously published dynamic intracellular Ca^2+^ cycling model (Shiferaw et al., 2003).

The model replicated experimentally-observed APD and Ca^2+^ transient alternans at rapid rates and accurately reproduced APD restitution curves obtained with dynamic or S1S2 pacing protocols. The model was then used to understand the anti-arrhythmic effects of inhibiting Ca^2+^-dependent *I*_Ca,L_ inactivation by overexpression of a mutant Ca^2+^-insensitive calmodulin in rabbit ventricular myocytes. The model was able to reproduce the experimentally-observed flattening of the APD restitution curve and the prevention of APD and Ca^2+^ transient alternans with altered *I*_Ca,L_ kinetics, which in two-dimensional (2D) tissue simulations prevented spatially discordant alternans and spiral wave breakup (Mahajan et al., 2008a).

The Shannon and Mahajan models, while similar in many respects, generate different outputs, especially for frequency-dependent responses. These two models were compared by Romero et al. through a systematic sensitivity analysis of AP, Ca^2+^, and Na^+^ dynamics, as well as rate dependence on variations in principal ionic currents’ conductance and kinetics (Romero et al., 2011). Simulations demonstrated that for both models APD is modified by changes in most repolarising K^+^ currents, AP triangulation is regulated primarily by *I*_K1_, and APD rate adaptation and [Na^+^]_i_ rate dependence are influenced mostly by *I*_NaK_. Steady-state [Ca^2+^]_i_, APD restitution properties and [Ca^2+^]_i_ rate dependence are also strongly dependent on *I*_NaK_, as well as *I*_Ca,L_ and *I*_NaCa_, although the relative role of these currents is strongly model dependent. Overall, simulations using both models agreed with many experimentally-reported electrophysiological characteristics in rabbit, however it was shown that the Shannon model reproduces rabbit electrophysiology more accurately at normal pacing rates, while the Mahajan model is more accurate at faster rates.

#### 1.2.4 Update of the Shannon model by Morotti et al

The most recently developed rabbit-specific ventricular model is an update of the Shannon model by Morotti et al., with the aim of improving the formulations of *I*_Ca,L_ and SR Ca^2+^ release (Morotti et al., 2012). In the model, *I*_Ca,L_ was altered to include experimentally-observed weak Ba^2+^-dependent inactivation and a greater efficacy in triggering SR Ca^2+^ release at negative membrane potential (*V*_m_), while SR Ca^2+^ release was modified to reproduce the experimentally measured *V*_m_ dependence of excitation-contraction gain. In the model, under physiological conditions *I*_Ca,L_ inactivates predominantly *via* Ca^2+^-dependent inactivation, controlled mostly by SR Ca^2+^ release during the initial phase of the AP, and by Ca^2+^ from *I*_Ca,L_ for the remainder, greatly outweighing voltage-dependent inactivation. As a result, simulations of impaired Ca^2+^-dependent inactivation predicted prolongation of APD and increased Ca^2+^-triggered excitation, providing novel insight into potential mechanisms of early and delayed after-depolarisations (EADs and DADs, respectively).

### 1.3 Multi-cellular ventricular simulations including cellular heterogeneity

Beyond the plethora of single cell studies investigating mechanisms underlying physiological and pathological electrophysiological function and potential molecular therapies, rabbit-specific cardiac cell models have been incorporated into anatomically-detailed and biophysically-detailed two- and three-dimensional multi-cellular simulations to study the importance of cellular heterogeneity on excitation and conduction and the induction and sustenance of arrhythmias. Sinoatrial node cell models (combined with atrial and fibroblast models) have been used to understand integrated behaviour of the complex, heterogeneous pacemaker region (Lang et al., 2011, Oren and Clancy, 2010, Zhang et al., 2001), while atrial models have been used to understand the role of anatomical and functional heterogeneity of the atria in the genesis of re-entrant arrhythmias and associated electrocardiogram (ECG) patterns (Aslanidi et al., 2009, Campos et al., 2013).

For the ventricles, the Puglisi-Bers model (Puglisi and Bers, 2001) has been used to investigate how mutation of the KCNQ1-G589D gene (associated with long-QT syndrome) may lead to ventricular arrhythmias during sympathetic stimulation in a computational rabbit ventricular wedge (Saucerman et al., 2004). It was found that while KCNQ1-G589D mutation alone had no effect, when coupled with β-adrenergic stimulation it resulted in QT prolongation and transient after-depolarisations, which amplified tissue heterogeneities, elevating transmural dispersion of repolarisation and creating T-wave abnormalities on the simulated ECG.

The Shannon model (Shannon et al., 2004) has also been used in rabbit ventricular wedge simulations for *in silico* prediction of pharmacological effects on the QT interval, which, when combined with concentration-effect data for block of *I*_Na_, *I*_Ca,L_, *I*_Kr_, and the slow delayed rectifier K^+^ (*I*_Ks_) currents, proved to be a highly accurate, sensitive, and specific assay for arrhythmic risk (Beattie et al., 2013).

The Mahajan model (Mahajan et al., 2008b) has been used more extensively in multi-cellular simulations. It has been used: to examine AP rise times and conduction velocity as a depolarising wavefront approaches the epicardial surface (Kelly et al., 2013); to assess how the electrophysiology of viable surface layers during isolated tissue superfusion are affected by pathological processes occurring in adjacent, poorly oxygenated tissue (Campos et al., 2012); to estimate how many contiguous susceptible myocytes are required for EADs and DADs to overcome electrotonic source-sink mismatch to trigger premature ventricular excitation (Xie et al., 2010); and to reproduce the timing distribution of spontaneous Ca^2+^ release *via* ryanodine receptors (sparks) and key features of the resulting Ca^2+^ waves and DADs (Chen et al., 2011).

The Mahajan model has further been employed in a 2D anatomic model of the rabbit ventricles with a simplified His-Purkinje system (including heterogeneous heart rate thresholds for DAD-induced bigeminy, an arrhythmia in which each normal beat is immediately followed by an ectopic beat) to evaluate the “ping pong” model of reciprocating bigeminy and bidirectional ventricular tachycardia (Baher et al., 2011) and in a 2D ventricular tissue model to determine how spiral waves respond to β-adrenergic stimulation and transition from ventricular tachycardia to fibrillation (Xie et al., 2014). Finally, it has been inserted into a model of the rabbit right ventricular wall to elucidate mechanisms of low-voltage cardioversion (Rantner et al., 2013) and into a rabbit ventricular slice model to investigate the role of the coronary vasculature in defibrillation (Bishop et al., 2010, Bishop et al., 2012).

### 1.4 Modelling of acute ischemia

The study of electrophysiological disturbances leading to arrhythmias due to heterogeneity caused by acute ischemia is one area in particular where rabbit-specific computational modelling has provided valuable insight (although in some cases, while rabbit-specific geometries were used, the underlying cellular models were in fact developed for other species) (Jie et al., 2010, Jie and Trayanova, 2010, Li et al., 2006, Michailova et al., 2007, Rodriguez et al., 2006a, Rodriguez et al., 2004, Rodriguez et al., 2006b, Tice et al., 2007).

Acute ischemia is a major cause of sudden cardiac death (Rubart and Zipes, 2005), thought to account for 80% of all sudden deaths without a prior history of heart disease (Myerburg et al., 1997). This is due to deadly ventricular arrhythmias (John et al., 2012), caused by well-described changes in cardiomyocyte AP characteristics (decreased AP upstroke velocity, amplitude, and APD and increased resting *V*_m_, *V*_rest_, and refractoriness) (Carmeliet, 1999). Combined, these cell-level alterations can lead to a variety of tissue-level electrical disturbances, including premature ventricular excitation, EADs and DADs, and re-entrant tachyarrhythmias (ventricular tachycardia or fibrillation) (Janse and Wit, 1989).

At the subcellular level, AP changes are largely due to altered ion concentrations (increased intracellular Na^+^, Ca^2+^, and extracellular K^+^, and decreased extracellular Na^+^), decreased intracellular ATP levels, intracellular pH, and *I*_Na_ and *I*_Ca,L_, and activation of the ATP-inactivated K^+^ current (*I*_K,ATP_) (Carmeliet, 1999). However, the cellular response to these ischemia-induced subcellular effects varies within and between subjects. At the same time, ischemia is a dynamic process, with changes progressing in time from its onset (Carmeliet, 1999). The result is a widely varying electrophysiological profile and associated arrhythmic risk.

While there has been relative success in investigating the contribution of ischemia-induced changes in cellular electrophysiology to arrhythmogenesis by experimental means (Janse and Wit, 1989), studying the critical contribution of regional (especially transmural) electrophysiological heterogeneity has proven difficult. This is another area where rabbit-specific computational models have been effectively employed to address experimental limitations.

Tice et al. developed a 2D cross-sectional electrophysiological model of the regionally-ischemic rabbit ventricles, including a central ischemic zone, surrounded by an ischemic border zone with transmural gradients of increased extracellular K^+^ concentration ([K^+^]_o_), decreased *I*_Na_ and *I*_Ca,L_, and *I*_K,ATP_ activation (Tice et al., 2007). Varying degrees of ischemia (representing progression from 2 to 10 min after coronary artery occlusion) were simulated, with premature stimulation applied over a range of coupling intervals to induce re-entry. It was demonstrated that the presence of an ischemic border zone and a transmural gradient in *I*_K,ATP_ activity was critical for sustained arrhythmias, by increasing dispersion of refractoriness and conduction velocity in this region, thus creating a substrate for re-entry that increased in severity with time post-occlusion.

This has since been followed up by Jie et al. in a study using a three-dimensional electromechanical model of the rabbit ventricles, which included regionally heterogeneous ischemia-induced changes in electrophysiological and mechanical function (Jie et al., 2010). Results from this investigation revealed a critical contribution of heterogeneous mechanical activity to both the origin of and substrate for arrhythmogenesis (Quinn, 2014), as mechanically-induced depolarisation resulted in premature ventricular excitation originating in the endocardium of the ischemic border, while mechanically-induced DAD-like events contributed to the formation of re-entry by further decreasing local excitability, causing extended conduction block lines and slowed conduction in the ischemic region.

Rabbit-specific computational modelling has also been used to demonstrate the effects of ischemic heterogeneity in the efficacy of anti-arrhythmic interventions. Rodríguez et al. used a three-dimensional electrophysiological model of the rabbit ventricles to understand defibrillation failure during the first 10 min of acute regional ischemia (Rodriguez et al., 2006a). By applying monophasic electric shocks over a range of coupling intervals, it was demonstrated that ischemia-induced electrophysiological heterogeneity did not alter the upper limit of vulnerability to shock-induced arrhythmias, as virtual electrode polarisation and post-shock behaviour remain unaffected. However, ischemia did result in a widening of the vulnerable window, due to increased likelihood of re-entrant circuits caused by conduction slowing and enhanced post-shock dispersion of refractoriness in the ischemic region.

Yet, while the importance of heterogeneity for arrhythmogenesis during acute ischemia is now clear, little is known about the importance of variability in the cellular response to ischemia. As is the case for arrhythmia mechanisms in most settings, current understanding of ischemia-induced arrhythmias is based largely on experimentally-derived mean responses, averaged across numerous subjects, and associated computational studies that represent a ‘typical’ case. This approach results in an inability to account for observations that may depend on the presence of normal physiological variability.

### 1.5 Modelling variability using populations of models

Whereas traditionally ventricular cell models have been constructed based on specific parameter values for ionic and cellular properties, extensive variability is reported experimentally from the ionic to whole organ level (Pueyo et al., 2011). Several computational modelling frameworks have been developed to study the implications of variability in cardiac electrophysiology (as reviewed previously (Muszkiewicz et al., 2016, Sarkar et al., 2012)). One important source of variability is the maximum conductance or permeability of each ionic current (the product of the channel unitary conductance and the number of channels for each current), which has traditionally been modelled as a constant parameter, but that is in fact variable (Pueyo et al., 2011). Recent papers have highlighted the large variety of sources of variability in ionic properties, not only due to inter-individual genetic differences, but factors such as hormones (Nakamura et al., 2007, Shuba et al., 2001), circadian rhythms (Jeyaraj et al., 2012), and nutrients (Erickson et al., 2013). These important findings call for novel approaches that allow investigating variability in phenotypes that arise from a large number of ionic profiles.

One such approach is experimentally-calibrated populations of cardiac cell models, which capture observed variability, often at the action potential level, while considering a wide range of ionic profiles to investigate their importance in reproducing cellular phenotypes in various (patho-)physiological settings (Muszkiewicz et al., 2016). Briefly, human-specific atrial myocyte models have revealed contributions of intrinsic and extrinsic factors to the range of APs recorded from similar subjects in sinus rhythm (Muszkiewicz et al., 2014) and mechanisms underlying inter-subject differences in early repolarisation and APD in patients with atrial fibrillation (Sanchez et al., 2014). Human-specific ventricular myocyte models have been used to identify common ionic mechanisms contributing to EADs in patients with hypertrophic cardiomyopathy (Passini et al., 2016), determine mechanisms responsible for voltage and Ca^2+^ alternans in patients undergoing cardiac surgery (Zhou et al., 2016), and to predict the relative importance of various transmembrane currents in determining susceptibility to drug-induced repolarisation abnormalities (Britton et al., 2014).

The rabbit-specific Purkinje cell model developed by Corrias et al. (2011) has similarly been used to predict ranges of APD prolongation with pharmacological block of *I*_Kr_ (Britton et al., 2013). More recently, Gemmell et al. focused specifically on developing and evaluating two experimentally-calibrated populations of rabbit-specific computational ventricular AP models (based on the Shannon (Shannon et al., 2004) and Mahajan (Mahajan et al., 2008b) models) that reproduce physiologic variability of repolarisation (Gemmell et al., 2014). In that study it was found that experimentally-observed intercellular variability of the rabbit ventricular AP could be reproduced by a population of computational models that includes large variations in currents’ conductance important for repolarisation.

As a follow up to that study, we have investigated the electrophysiological response of these two populations of rabbit models to acute ischemia. The primary hypothesis was that under ischemic conditions, normal physiological variability will be exaggerated, which may help explain inter-subject differences in ischemic responses. A systematic exploration of the response of the model populations to varying degrees of ischemia and individual ischemic changes was performed, to investigate their individual and combined effects on AP dynamics. Results demonstrate complex interactions between variability in ion channel function and ischemic factors, which combine to produce large variability in the AP response to ischemia. Overall, this work represents a step towards an improved understanding of the role of physiological variability in ischemia and for the incorporation of experimentally-observed variability into computational simulations for understanding arrhythmic risk.

## 2 Material and methods

### 2.1 Populations of rabbit-specific ventricular AP models

Two experimentally-calibrated, biophysically-detailed populations of rabbit-specific computational ventricular AP models that produce physiologic values of APD at three cycle lengths (400, 600, and 1000 ms) (Gemmell et al., 2014) were adapted for simulations of ischemia. Each population consists of an underlying set of governing equations combined with a parameter set that defines combinations of six independent currents’ conductance that produce a physiological AP (with the assumption that AP variability is primarily a result of differences in the relative magnitude of currents, rather than underlying current dynamics). The populations were originally constructed by uniformly sampling the parameter space, and then calibrated by constraining output parameters through comparison with experimental data; details regarding their development can be found in the related publication (Gemmell et al., 2014) (for methodological details in general, please see a comprehensive review on the subject (Muszkiewicz et al., 2016)). The underlying equations are those developed by Shannon et al. (2004) (including corrections (Shannon et al., 2012)) and Mahajan et al. (2008b) to simulate the AP of a rabbit ventricular epicardial myocyte, described above. Both models were originally downloaded from the CellML model repository (http://models.cellml.org/cellml), adapted by unclamping intracellular K^+^ concentration, and converted to C++ using the Cellular Open Resource (COR) software (http://cor.physiol.ox.ac.uk/) (Garny et al., 2009). The transmembrane currents with variable conductance (*g*) are: *I*_to_ (*g*_to_); *I*_Kr_ (*g*_Kr_); *I*_Ks_ (*g*_Ks_); *I*_K1_ (*g*_K1_); the *I*_Ca,L_ (*g*_Ca,L_); and *I*_NaK_ (*g*_NaK_). For the Shannon model the population consists of 1352 combinations of currents’ conductance, while the Mahajan population consists of 779 combinations (constrained by experimentally reported values of APD). Importantly, of the small animals, rabbit has cardiac electrophysiology most similar to human, and thus is a preferred model for experimental research and pharmacological testing (Nattel et al., 2008), providing established reference values for constraining models to a physiological range.

### 2.2 Simulation of acute ischemia

To simulate the effect of ischemic conditions on rabbit cellular electrophysiology, the effects of varying degrees of hypoxia, acidosis, and hyperkalemia on ionic properties were included in the simulations. In line with previous computational studies (Rodriguez et al., 2006b, Shaw and Rudy, 1997), we chose to consider changes with the greatest influence on the AP and refractory period, shown to be the most relevant for the establishment of re-entrant circuits (Dutta et al., 2016, Trenor et al., 2005). The electrophysiological effect of hypoxia was simulated using a previously reported formulation of *I*_K,ATP_ (Michailova et al., 2005), with the degree of activation set by multiplication of the current conductance by a scaling factor (*f*_K,ATP_). After 10 min of ischemia, *f*_K,ATP_ has been estimated to be on the order of 0.8%, resulting in a total channel conductance of 0.02088 mS/μF (Michailova et al., 2007) and approximately a 45% reduction in APD_90_ (Ferrero et al., 1996, Shaw and Rudy, 1997, Weiss et al., 1992). As total *I*_K,ATP_ conductance is the product of *f*_K,ATP_ and *g*_K,ATP_, *g*_K,ATP_ was set as 2.61 mS/μF to produce similar results. The effect of acidosis was simulated by inhibition of *I*_Na_ and *I*_Ca,L_, through multiplication of each current conductance by a scaling factor (*f*_inhib_). Hyperkalemia was simulated by increasing [K^+^]_o_. In addition to these well-established ischemic effects, [Na^+^]_i_ was increased (Noble, 2002) and *I*_NaK_ was reduced (Terkildsen et al., 2007), through multiplication of its current conductance by a scaling factor (*f*_NaK_). To approximate conditions during the first 10 min of ischemia, a linear increase in ischemic parameters was used. For simplicity, we refer to the different degrees of ischemic severity by time (values are given in Table 1), but highlight the fact that the time course is dependent on experimental conditions and differs across regions of the rabbit ventricles. Furthermore, three different final values of [K^+^]_o_ were considered (see Table 1), as well as the effect of varying levels of *I*_K,ATP_.

The combination of currents’ conductance that produced the closest APD to the mean experimentally reported values at all three pacing rates in our previous study was selected to represent control conditions (percentage change from original model values are given in Table 2). Cells were stimulated at a pacing cycle length of 600 ms with a 3 ms pulse and the stimulus current (*I*_stim_) set to 1.5 times the excitation threshold in control (an appropriate value of *I*_stim_ is an important consideration for simulations of ischemia, as refractory period depends on the stimulus strength (Sutton et al., 2000)). Simulations were run for 10 stimulated APs, performed using an ordinary differential equation solver with adaptive time-stepping (CVODE) and relative and absolute tolerances set to 10^−7^ and 10^−9^, respectively.

### 2.3 Analysis of ischemic effects on AP characteristics

For each simulation, the last stimulated AP was analysed. If the amplitude of the change in *V*_m_ in response to the last stimulation was less than 44 mV, this was not considered an AP – the stimulation was considered as to have failed to cause excitation and was excluded from further analysis.

Several commonly used measures of AP morphology were calculated: (i) *V*_rest_; (ii) maximum *V*_m_ (*V*_max_); (iii) maximum rate of *V*_m_ change (d*V*_m_/dt_max_); and (iv) APD at 90% repolarisation (APD_90_, measured as the time interval between the point of d*V*_m_/dt_max_ and the point when *V*_m_ was repolarised to 90%, *i.e.*, *V*_m_ ≤ *V*_rest_ + 0.1 × [*V*_max_ − *V*_rest_]). Effective refractory period (ERP, the minimum time after stimulation that a cell has regained excitability) was assessed from the product of the *I*_Na_ inactivation gates (*h* × *j*) (Pandit and Jalife, 2013, Romero et al., 2009, Tice et al., 2007, Trenor et al., 2007). A value > 0.012 was set as the threshold at which a cell has become re-excitable (Tice et al., 2007). All analyses were performed using MATLAB (R2011b, version 7.13.0.564; MathWorks, Nantick, MA).

### 2.4 Analysis of effects across the model populations

To assess the variability of measured values across the model populations, we calculated their variance and range. To visualise more complex effects across the model populations in the six-dimensional parameter space resulting from variation in the magnitude of currents’ conductance, we used alternative techniques. The first technique allows visualisation of the variability in the response of a measured value with each combination of currents’ conductance by the creation of a column plot in which each row represents a single combination of currents’ conductance and each column represents a different time point of ischemia. The second technique, known as clutter-based dimension reordering (which was originally developed for studies of variability in neuronal electrophysiology (LeBlanc et al., 1990, Peng et al., 2004, Taylor et al., 2006) and subsequently employed in our study of variability in ventricular repolarisation (Gemmell et al., 2014)) enables visualisation of the influence of currents’ conductance on measured values in a high-order parameter space by a linear projection to a lower dimensional space (typically of one- or two-dimensions). This projection is accomplished by transforming a given point in *n*-dimensions (with coordinates (*x*_1_, …, *x*_*n*_)) to a unique point in a lower dimension. For a one-dimensional space (with coordinate *x*_*i*_’), this is given by:

$$x_{i}^{\prime }=\sum_{i=1}^{n}\left(\left(x_{i}-1\right)\prod_{j=1}^{i-1}N_{j}\right)+1\text{,}$$

which returns a value between 1 and *N*, where *N* is the total number of data points (determined in each dimension by *N*_*i*_). Clutter-based dimension reordering can be conceptualised as taking ’slices’ from a higher dimensional space and arranging them in a lower dimension (much like slicing a cube and placing the resulting squares next to each other, only with slices taken in more than three dimensions, such that with continuous slicing the dimensionality of the space is iteratively reduced until it can be visualised, with each level presenting the effect of variation in a pair of currents’ conductance).

## 3 Results

### 3.1 Effect of ischemia on variability of APD

**Fig. 1:**
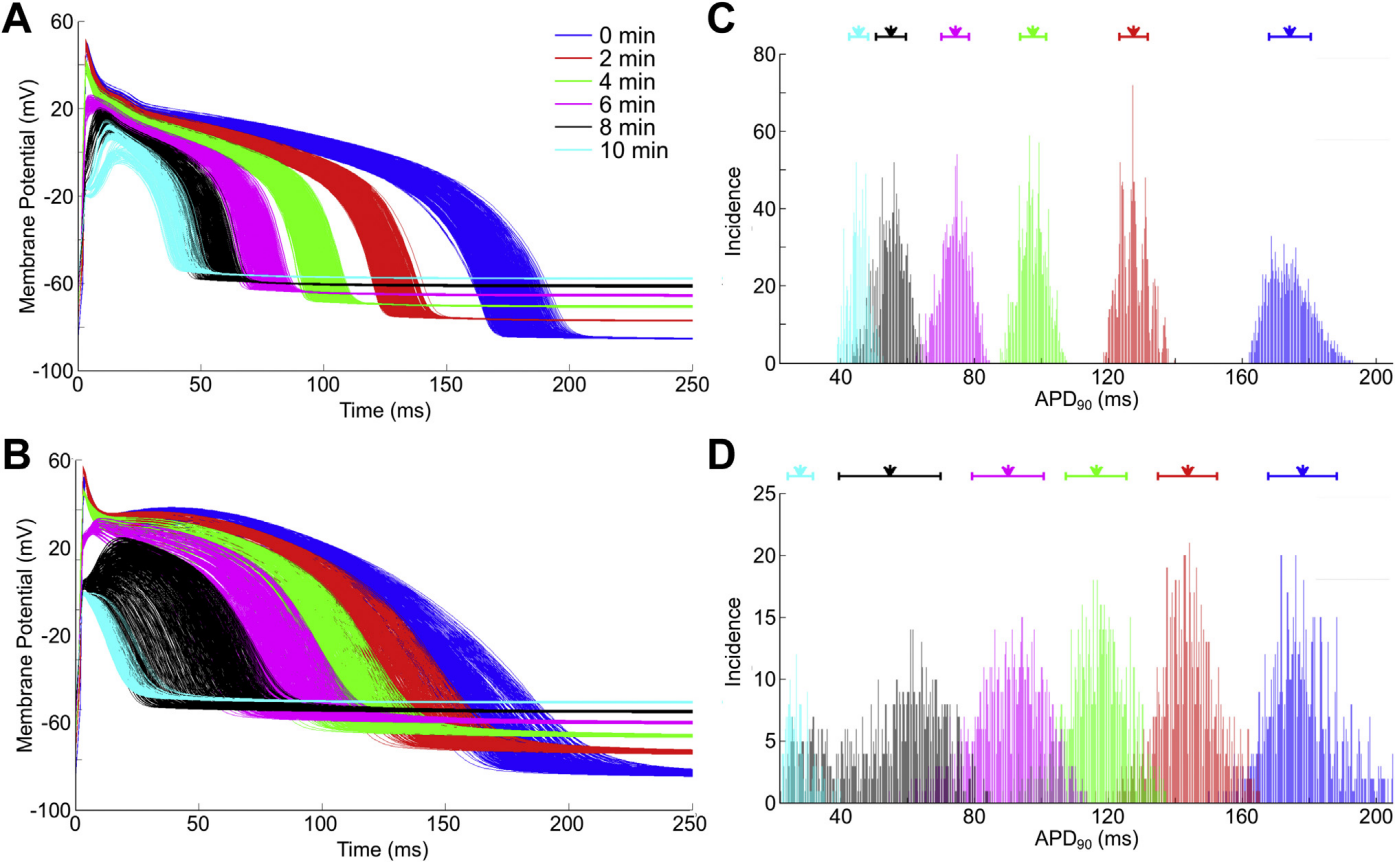
**Effect of increasing ischemic severity on action potential variability**. Action potentials from the Shannon (A) and Mahajan (A) model populations at different points during the first 10 min of ischemia, as well as histograms showing the distribution of action potential duration (APD_90_) in the Shannon (C) and Mahajan (D) populations. Arrows indicate mean values of APD_90_, with bars presenting standard deviation.

The APs from the two model populations at different points during the first 10 min of ischemia, along with histograms demonstrating the change in the distribution of APD_90_ are shown in Fig. 1. Both model populations reproduced the most prevalent (and well established) changes in AP characteristics reported to occur during acute ischemia in rabbit ventricles, namely an increase in *V*_rest_ and ERP, and a decrease in AP upstroke velocity, AP amplitude, and APD. Values are similar to those reported for rabbit isolated ventricular cells subjected to simulated ischemia (de Groot et al., 2003), in which APD_90_ was decreased by 92% following 20 min of ischemia. Thus, APD shortening was in line with experimental values, but changes took longer in the experiments than in our simulations, which were based on the time course of ionic changes reported in previous experimental and simulation studies. In contrast, APD shortening in our simulations and in isolated cell experiments were larger than those reported in rabbit isolated hearts and tissue preparations (Barrett et al., 1997, Behrens et al., 1997, Guo et al., 2012, Huang et al., 2004, Kurz et al., 1993a, Kurz et al., 1993b, Vermeulen et al., 1996, Weiss and Shine, 1982, Weiss et al., 1992). This suggests variability in the magnitude of *I*_K,ATP_ activation (the size of this current has a large effect on APD_90_), which will be investigated in Section 3.3.

Both model populations displayed similar changes in APD variability with increased ischemic severity (although overall, the variability of the Shannon model population was considerably lower than the Mahajan). There was an initial decline in both the variability and range of APD_90_ in the first 2 min of ischemia, followed by an increase between 2 and 8 min. This was followed by a sharp decline at 10 min, which was primarily due to the exclusion of a significant number of currents’ conductance combinations for which stimulation failed to excite the cell, as ERP increased to greater than the cycle length (650 [45%] for the Shannon and 613 [79%] for the Mahajan model populations).

### 3.2 Effects of individual ischemic parameters

**Fig. 2:**
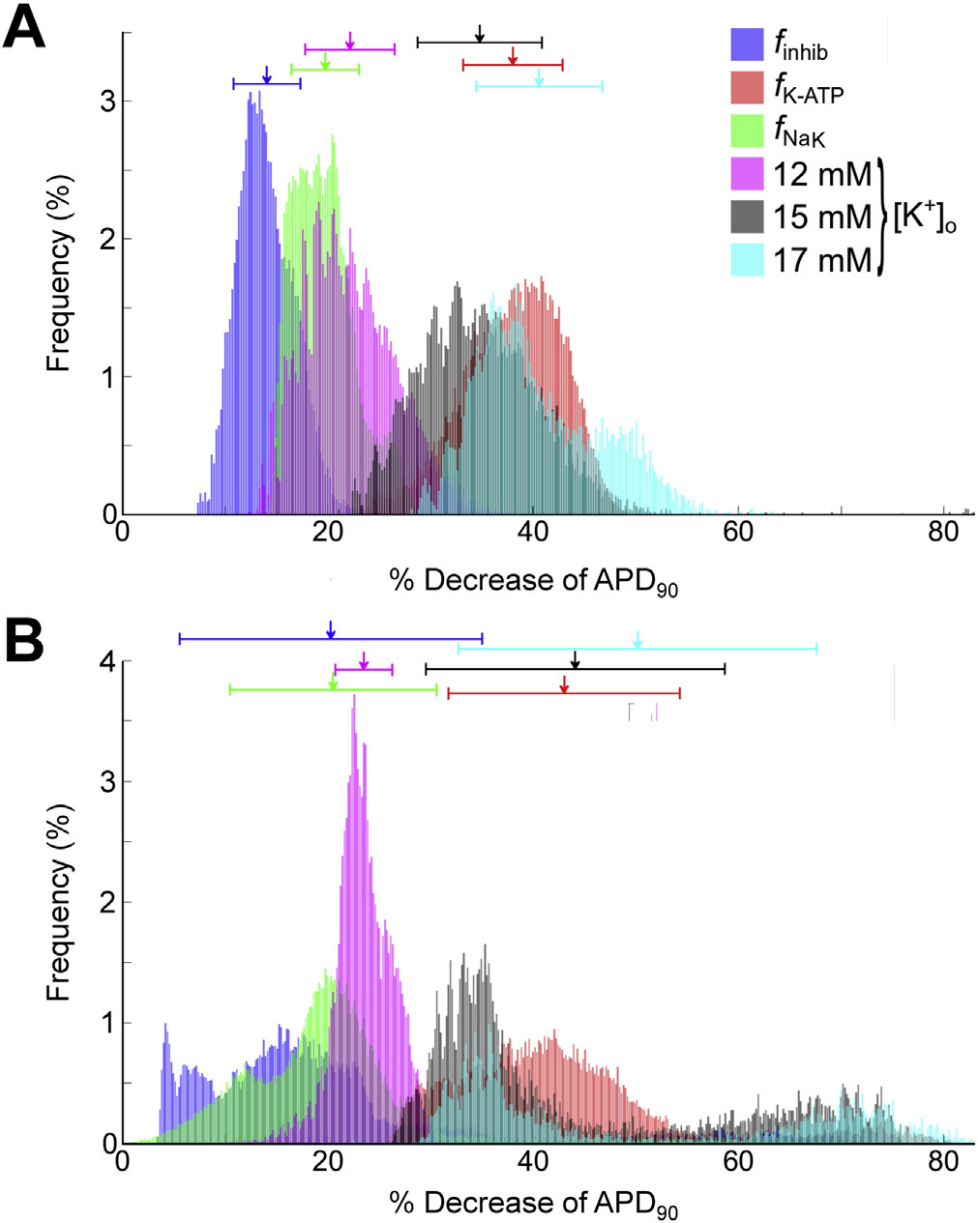
**Effect of change in individual ischemic parameters on action potential duration**. Histograms showing the percentage decrease of action potential duration (APD_90_) in the Shannon (A) and Mahajan (B) populations resulting from a change in individual ischemic parameters to their 10 min value. For each, the other ischemic parameters are set to all combinations of tested values. Arrows indicate mean values of APD_90_, with bars presenting standard deviation.

Fig. 2 presents histograms demonstrating the percentage decrease in APD_90_ resulting from a change in individual ischemic parameters to their 10 min value, with the other ischemic parameters set to all combinations of tested values. Both model populations demonstrate a similar relative importance of the various ischemic parameters on APD_90_. *I*_K,ATP_ activation (represented by *f*_K-ATP_) had the greatest mean effect in both populations, while inhibition of *I*_Na_ and *I*_Ca,L_ (*f*_inhib_) had the smallest effect, followed by reduced *I*_NaK_ (*f*_NaK_) and increased [Na^+^]_i_. The effect of hyperkalemia on APD_90_ increased with increasing [K^+^]_o_, such that at lower levels the effect was similar to reducing *I*_NaK_, but became as potent as *I*_K,ATP_ activation at higher levels. Simultaneous changes in the other ischemic variables caused a wide spread in values of APD_90_, which were much larger for the Mahajan than Shannon model (even though the mean values were similar).

### 3.3 Effect of varying I_K,ATP_

**Fig. 3:**
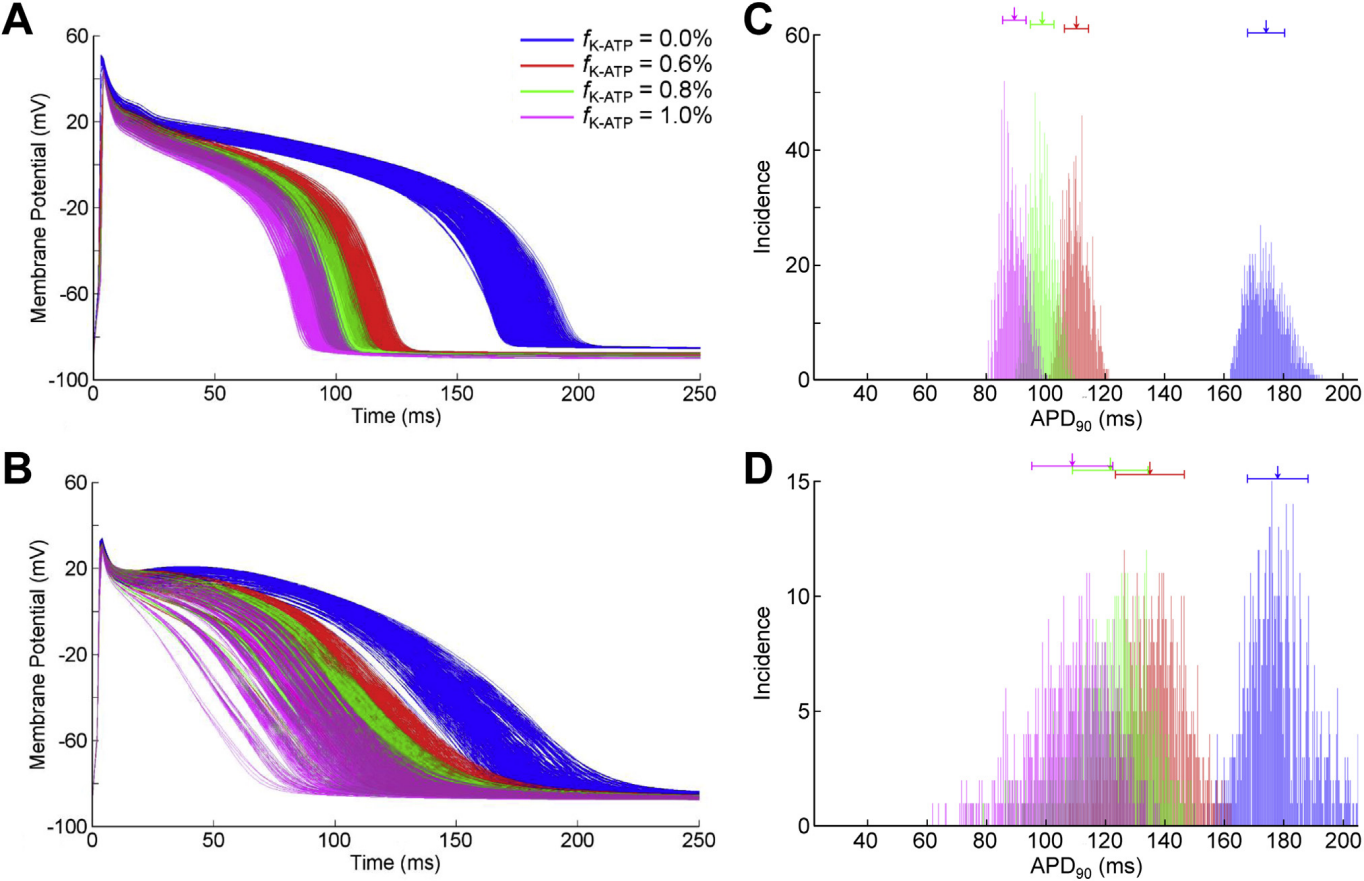
**Effect of increasing ATP-inactivated potassium current (*I***_**K,ATP**_**) conductance on action potential variability during control**. Action potentials from the Shannon (A) and Mahajan (B) model populations with different levels of *I*_K,ATP_ activation (*f*_K-ATP_) in control conditions, as well as histograms showing the distribution of action potential duration (APD_90_) in the Shannon (C) and Mahajan (D) populations. Arrows indicate mean values of APD_90_, with bars presenting standard deviation.

Fig. 3 presents APs for both model populations with varying *I*_K,ATP_ in control conditions, along with histograms showing the distribution of APD_90_. Increasing *I*_K,ATP_ shortens APD by increasing the negative slope, thus reducing the length of the AP plateau (a similar effect occurs under ischemic conditions; not shown). This is similar for both model populations, however the effect of increasing *I*_K,ATP_ on APD variability varies between the two. Both in control and during ischemia, increasing *I*_K,ATP_ decreases APD variability in the Shannon population. For the Mahajan population, however, APD variability is increased in control, while it is greatly decreased during ischemia (again, partly due to exclusion of some currents’ conductance combinations due to failed excitation with an increase in ERP to greater than the cycle length).

### 3.4 Effect of varying [K^+^]_o_

**Fig. 4:**
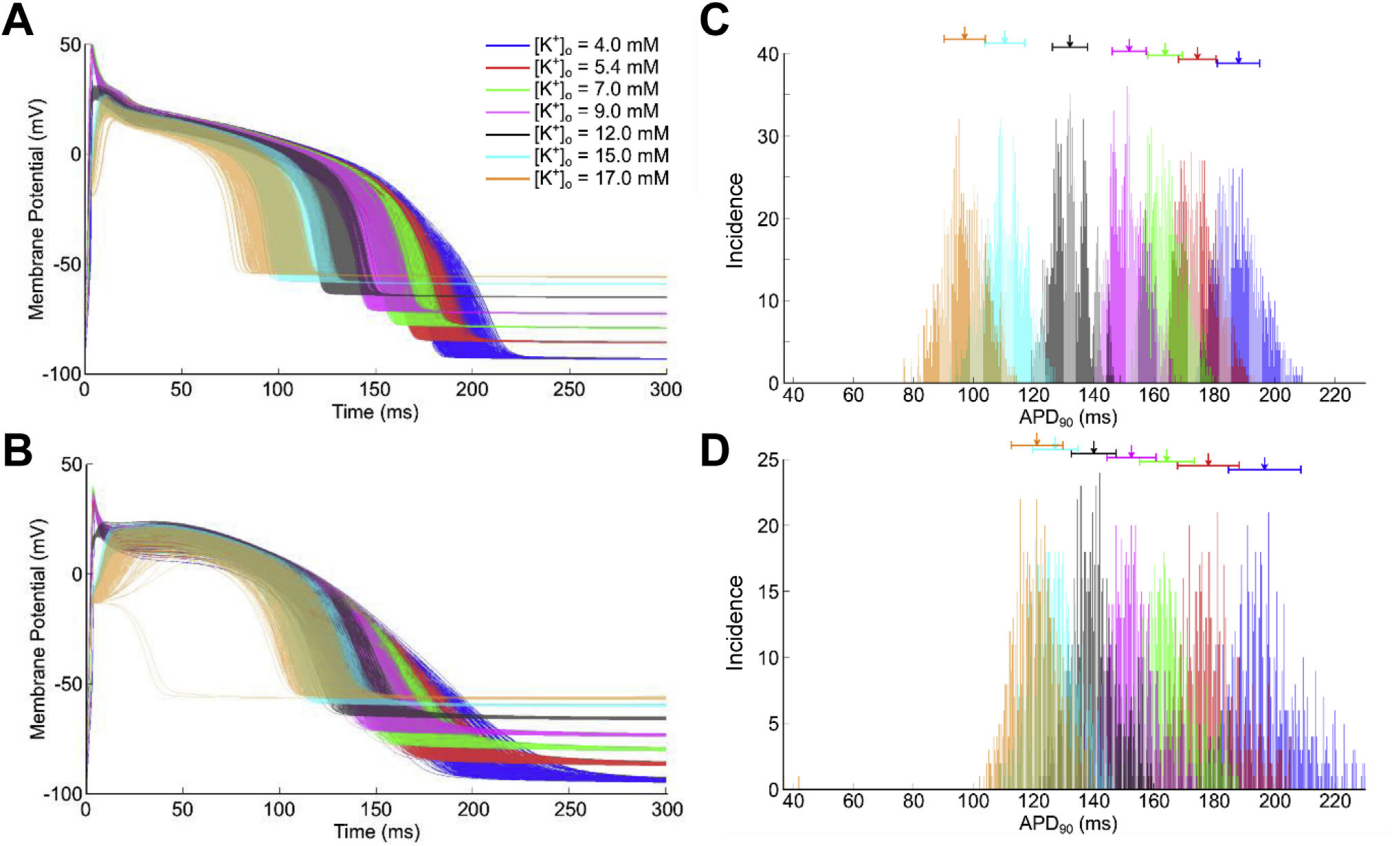
**Effect of increasing extracellular potassium concentration ([K**^**+**^**]**_**o**_**) on action potential variability during control**. Action potentials from the Shannon (A) and Mahajan (B) model populations with different levels of *I*_K,ATP_ activation (*f*_K-ATP_) in control conditions, as well as histograms showing the distribution of action potential duration (APD_90_) in the Shannon (C) and Mahajan (D) populations. Arrows indicate mean values of APD_90_, with bars presenting standard deviation.

Fig. 4 presents APs for both model populations with varying [K^+^]_o_ in control conditions, along with histograms showing the distribution of APD_90_. Increasing [K^+^]_o_ in control progressively decreases APD in both model populations, with little effect on variability. While the overall variability in APD is greatly increased in ischemia for the Mahajan population only, a similar reduction in APD with increasing [K^+^]_o_ is seen for both models, until excitation failure occurs at the most extreme value (not shown).

### 3.5 Effect of ischemia on variability of ERP

**Fig. 5:**
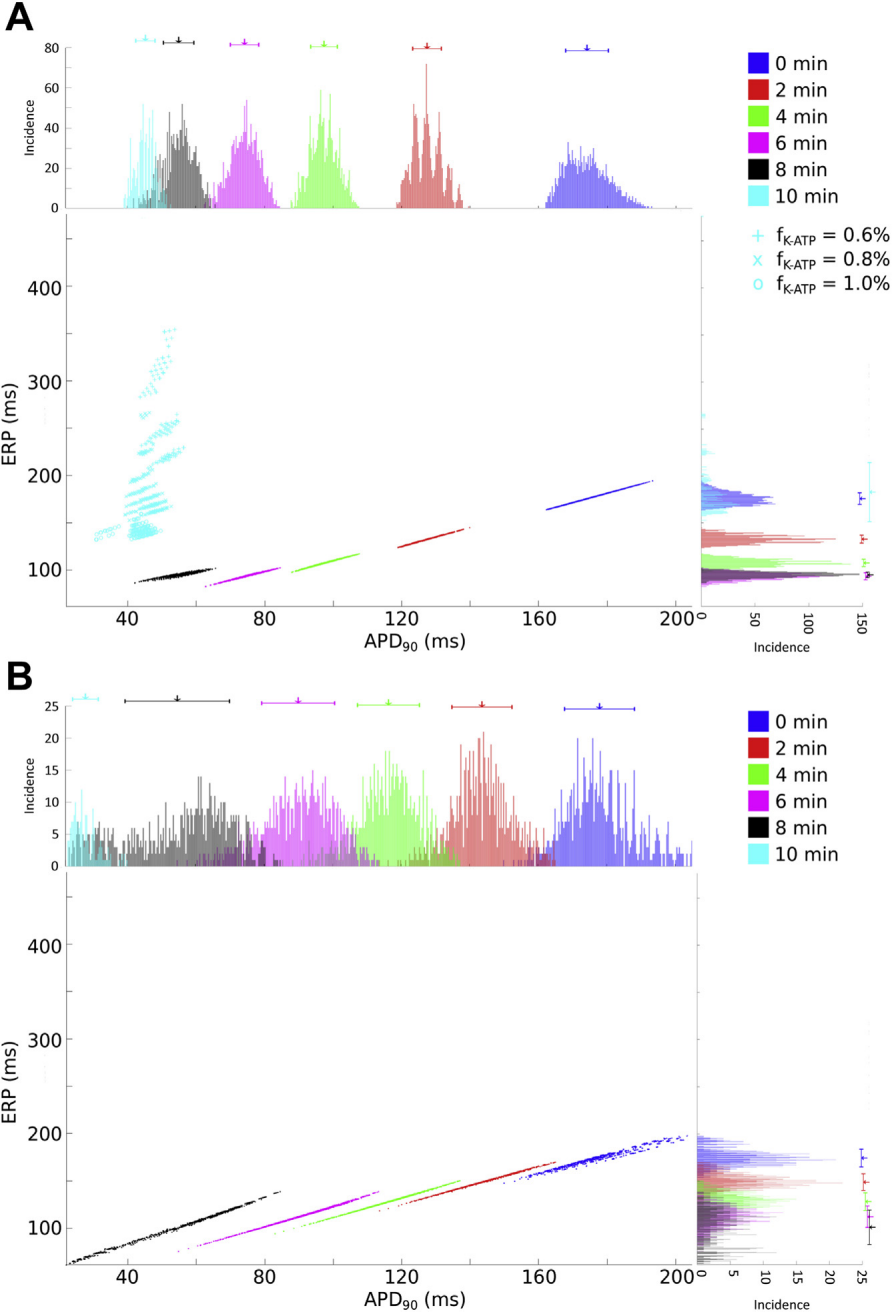
**Relationship between action potential duration (APD**_**90**_**) and effective refractory period (ERP) with increasing ischemic severity**. Scatter plots of APD_90_*versus* ERP at various stages of ischemia, and with increasing ATP-inactivated K^+^ current conductance (*f*_K-ATP_), for the Shannon (A) and Mahajan (B) model populations, along with histograms showing the distributions of APD_90_ and ERP.

Fig. 5 shows scatter plots of the relationship between APD_90_ and ERP at various stages of ischemia for each model population. There was a 1:1 correlation between the two variables for the first 6 min of ischemia. However, with more severe ischemia, mean ERP and its variability began to increase, resulting in a loss of the direct correlation between ERP and APD, an effect that was exasperated by decreasing *I*_K,ATP_, as shown for the Shannon population at 10 min of ischemia (not shown for the Mahajan population as ERP was in most models longer than the stimulation period).

**Fig. 6:**
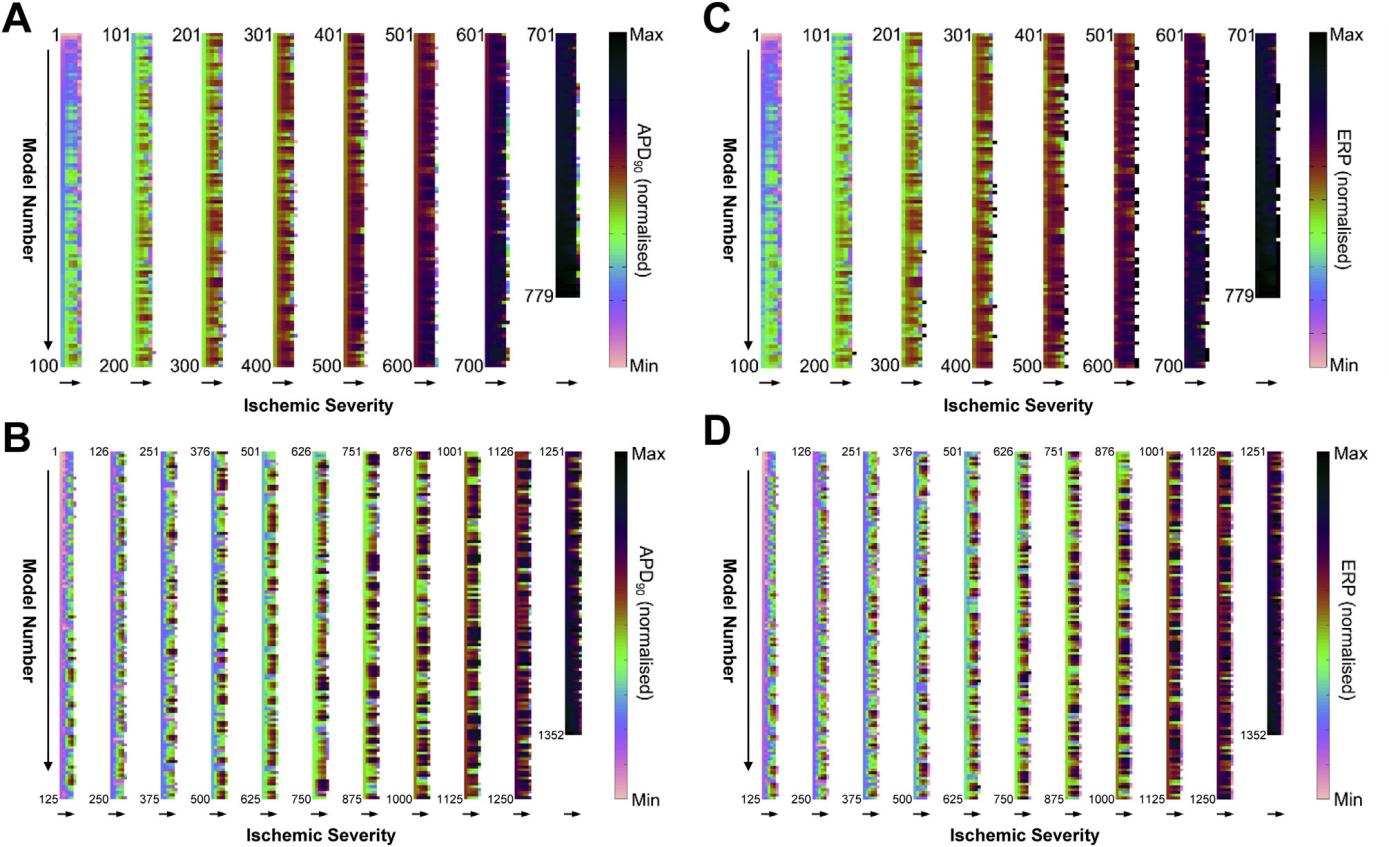
**Evolution of action potential duration (APD**_**90**_**) and effective refractory period (ERP) with increasing ischemic severity for each combination of currents’ conductance in the model populations**. Column plots of normalised APD_90_ and ERP (divided into multiple bars of 100 rows each) with all combinations of currents’ conductance (each row represents a single combination) in the Shannon (A,C) and Mahajan (B,D) model populations at different points during the first 10 min of ischemia (each column represents a different time point). The models are arranged in sequence of APD_90_ value under control conditions.

The evolution of APD_90_ and ERP with increasing ischemia for each combination of currents’ conductance in the two populations can be seen in Fig. 6. The column plots show increasing variability in the response of APD_90_ and ERP in each model with increasing ischemic severity.

**Fig. 7:**
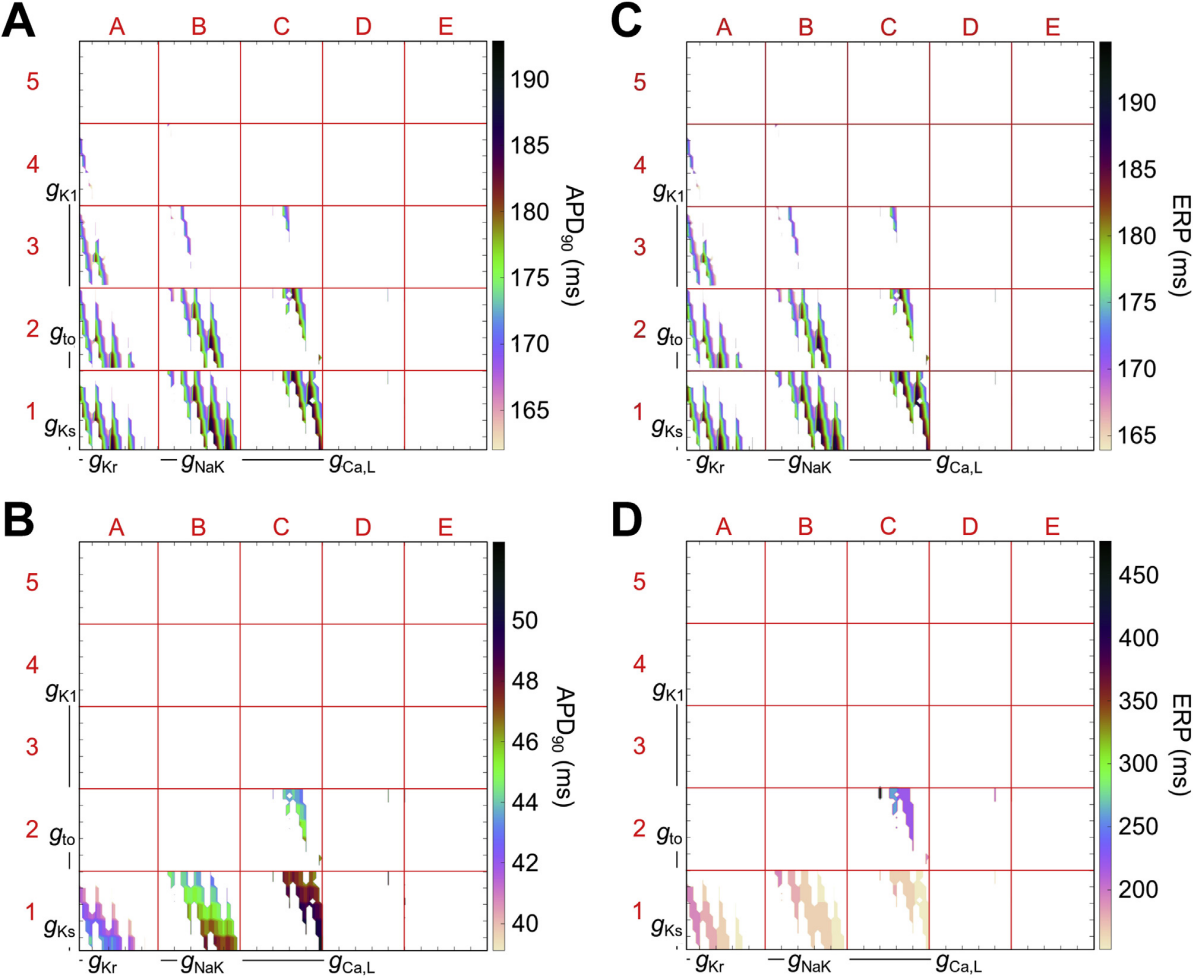
**Sensitivity of action potential duration (APD**_**90**_**) and effective refractory period (ERP) to changes in currents’ conductance during control and ischemia in the Shannon population**. Clutter-based dimension reordering images of APD_90_ and ERP with all viable combinations of currents’ conductance in the Shannon model population in control conditions (A,C) and at 10 min of ischemia (B,D).

Fig. 7 shows 2D plots of the six-dimensional parameter space for APD_90_ and ERP in control and ischemic conditions for the Shannon population, generated by clutter-based dimension reordering (the Mahajan population is too sparse for dimensional stacks to be useful). This allows investigation of the sensitivity of these measurements to changes in currents’ conductance under the two conditions. In control, both APD_90_ and ERP are most sensitive to changes in *g*_Kr_ (demonstrated by the rapid change in value at the smallest scale in the x-direction, which represents *g*_Kr_), while they are relatively insensitive to changes in *g*_Ca,L_ and *g*_K1_ (little difference between rows or columns at the largest scale). With ischemia, the relative sensitivity of APD_90_ and ERP to currents’ conductance changes. For APD, *g*_Ca,L_ and *g*_K1_ have a greater influence and *g*_to_ becomes relatively important, while the effect of changes in *g*_Kr_ declines. ERP, however, responds differently (further highlighting the loss of correlation between ERP and APD), as while increasing *g*_K1_ decreased APD_90_, it increased ERP (compare C1 and C2) and the increased importance of *g*_to_ did not apply.

## 4 Discussion

In this study we examined the electrophysiological response to acute ischemia of two populations of rabbit-specific computational ventricular AP models that reproduce experimentally-observed variability of repolarisation under normal conditions. Simulations involved varying degrees of ischemia and changes in individual ischemic parameters to assess their individual and combined effects on AP dynamics. Overall, the results demonstrate complex interactions of underlying population variability in currents’ conductance and ischemic factors in both model populations, which combine to produce large variability in the AP response. This has potentially important implications for arrhythmias during ischemia, as increased cellular variability will contribute to increased tissue heterogeneity, which increases the potential for sustained re-entrant activity, and may also explain varying individual susceptibility to ischemia-induced arrhythmias.

### 4.1 Changes in APD variability during ischemia

Our results indicate that variability in the response of repolarisation to acute ischemia is not linear. In both model populations there was a decline in variability of APD in the first 2 min of ischemia, suggesting that there is no contribution of changes in cellular variability to an arrhythmic substrate (heterogeneous APD) during this period. This agrees with the experimentally-measured low susceptibility to arrhythmias immediately after coronary artery occlusion (Janse and Wit, 1989, Kaplinsky et al., 1979). This decrease in APD variability was followed by an increase between 2 and 8 min. In tissue, this could contribute to tissue heterogeneity and an increase in the substrate for re-entrant arrhythmias (again in line with experimental results (Janse and Wit, 1989, Kaplinsky et al., 1979)), especially if unmasked by reduced cellular coupling (Pueyo et al., 2011) during later phases of ischemia (De Groot and Coronel, 2004) or with disease (Coronel et al., 2013, Vermeulen et al., 1996). At 10 min, ERP for many of the models in the two populations increased beyond the cycle length, resulting in failed excitation¸ which in tissue would further increase arrhythmia susceptibility by causing regional conduction block (Coronel et al., 2012).

There is experimental evidence to support differing electrophysiological responses and varying loss of excitability with ischemia between cell populations from the same heart. Using canine endocardial, epicardial, papillary muscle, and Purkinje fibre preparations subjected to simulated ischemia, Gilmour and Zipes demonstrated a greater reduction of AP amplitude, d*V*/dt_max,_ and prolongation of activation times after 10 min of ischemia in epicardial tissue compared to endocardial tissue or Purkinje fibres, despite similar changes in *V*_rest_ (Gilmour and Zipes, 1980). After 15 min, only 3 of 18 epicardial and 5 of 16 papillary preparations were excitable, whereas 14 of 16 endocardial and 13 of 13 Purkinje preparation were still responsive.

When we looked at the effect of individual ischemic parameters on the response of our model populations to ischemia, the extent of APD shortening was most influenced by the actions of hypoxia (activation of *I*_K,ATP_) and hyperkalemia (increase in [K^+^]_o_). While individually increasing *I*_K,ATP_ or [K^+^]_o_ resulted in different effects in the two model populations (decreased or no change in APD variability in the Shannon population, but increased APD variability with both in the Mahajan population), changes in either of these parameters combined with changes in other ischemic parameters greatly enhanced APD variability. Thus, the cell populations did not respond to ischemia in an easily predictable manner, as changes in APD depended on interaction of multiple components of the ischemic substrate with the underlying electrophysiological variability.

A contribution of the interaction of ischemic parameters to the electrophysiological response has been demonstrated by Morena et al. in isolated perfused pig hearts, where effects of hypoxia, hyperkalemia, and acidosis on AP amplitude, d*V*/dt_max_, *V*_rest_, and local activation varied drastically when applied in different combinations (Morena et al., 1980). Similarly, Rodríguez et al. have shown computationally that the integrated effect of *I*_K,ATP_ activation, ischemic Na^+^ inward current, and Na^+^-K^+^ pump inhibition is needed to replicate effects seen in ischemia (Rodriguez et al., 2002). This would suggest that differences in spatial gradients in ischemic parameters in tissue (that have been measured experimentally (Coronel, 1994, Coronel et al., 1988, Schaapherder et al., 1990, Wilensky et al., 1986)) could cause interactions that may enhance underlying cellular variability and contribute to pro-arrhythmic APD heterogeneity.

### 4.2 Changes in ERP variability during ischemia

We also examined the relationship between the distributions of APD and ERP across the two model populations as ischemia progressed. At first, values of APD and ERP were close to identical. As the cell populations became more ischemic, however, the mean value of ERP and its variability began to increase with the development of post-repolarisation refractoriness (another significant arrhythmic mechanism in ischemia (Coronel et al., 2012)). Interestingly, ERP became longer and more variable with a decrease in *I*_K,ATP_, reflecting a loss of the strong repolarising effect of this current (Zhang et al., 2010). A loss of a direct 1:1 relationship between APD and ERP during the initial ischemic period has been described in the Langendorff-perfused porcine heart (Coronel et al., 2012), as well as in patients undergoing coronary artery surgery (Sutton et al., 2000). The evolution of APD and ERP specifically in each model of the population, on the other hand, was revealed by the use of column plots, which demonstrated a high degree of variability in the response of each model to increasing ischemic severity.

### 4.3 Importance of currents’ conductance interactions

The benefits of a population of models approach as a tool to explore the implications of ionic variability is enhanced by use of specialised techniques for visualisation of multi-dimensional parameter spaces, such as clutter-based dimension reordering (Gemmell et al., 2014, LeBlanc et al., 1990, Peng et al., 2004, Taylor et al., 2006), which help reveal interaction of individual model parameters (in our case currents’ conductance), as well as their relative importance for measured values (in our case APD_90_ and ERP). For our model populations, both APD and ERP are most sensitive to changes in *I*_Kr_ in control, however the relative influence of this current is reduced as ischemia progresses (supporting previous experimental findings (Carmeliet, 1999)). At the same time, the initially minor influences of *I*_Ca,L_ and *I*_K1_ in control, along with *I*_to_, become relatively important in determining APD shortening in ischemia, while *I*_K1_ causes an increase in ERP (contributing to the loss of the APD-ERP relationship).

### 4.4 Methodological considerations and potential limitations

In this study, we were interested in the influence of underlying physiological cellular variability on the electrophysiological response to the primary effects of acute ischemia. As this was a single cell study, tissue-level effects were not explicitly investigated. Instead, cell-level changes in variability of APD and ERP under varying degrees of hypoxia, acidosis, and hyperkalemia were assessed. While these factors represent the range of conditions that are present across the ischemic border during regional ischemia (Coronel, 1994, Coronel et al., 1988, Schaapherder et al., 1990, Wilensky et al., 1986), in tissue, cellular coupling may mitigate the electrophysiological heterogeneity seen in single cells (Coronel, 1994, Coronel et al., 1988, Schaapherder et al., 1990, Wilensky et al., 1986). However, as significant cellular uncoupling occurs during acute ischemia (De Groot and Coronel, 2004), the observed increase in cellular variability with increasing severity of ischemia may indeed have important tissue-level implications for arrhythmogenesis (Dutta et al., 2016). Studying the tissue-level importance of enhanced cellular variability during acute regional ischemia should be the target of future studies.

At the cellular level, we employed previously utilised approaches for modelling the most prominent effects of acute ischemia on cellular electrophysiology. These resulted in the most prevalent changes in AP characteristics (increased *V*_rest_ and ERP, and decreased AP upstroke velocity, AP amplitude, and APD) reported for rabbit ventricles (Barrett et al., 1997, Behrens et al., 1997, Guo et al., 2012, Huang et al., 2004, Kurz et al., 1993a, Kurz et al., 1993b, Vermeulen et al., 1996, Weiss and Shine, 1982, Weiss et al., 1992), with values similar to those measured in rabbit isolated ventricular cells subjected to simulated ischemia (de Groot et al., 2003). The ischemic model, however, was based on data from several species (not solely from rabbit) and did not include all aspects of ischemia (such as more complex pH regulation and effects of protons on Ca^2+^ buffering (Terkildsen et al., 2007)). Thus, responses and the degree of enhanced cellular variability with acute ischemia observed in our simulations may differ from what occurs in actual cell populations, which may partly explain the differences in the time course of changes between our simulations and previous experiments.

Differences between computational and experimental responses may be further impacted by difficulties of directly parameterising computational models with data from experimental studies, especially in a species-specific manner. For studies of acute ischemia, there are currently insufficient data reporting changes in ionic currents’ conductance and kinetics for the rabbit (or for that matter, any other species). Moreover, ischemia is a dynamic process, with highly variable effects depending on experimental aspects impacting its degree and timing. For instance, differences in environmental control, especially temperature, can have large impacts on APD (a decrease in temperature increases APD (Lab and Woollard, 1978)), while diffusion of oxygen from perfusate or air surrounding isolated heart and tissue preparations can lead to non-ischemic (or least less ischemic, which will slow changes) cells at the surface (Wilensky et al., 1986). Experimental conditions, therefore, can have a large impact on AP recordings. Since measurements in whole heart are typically taken from the ventricular surface, this may possibly explain the smaller degree of APD shortening measured in isolated hearts exposed to room air *versus* isolated cells. The region from which measurements are taken (or cells isolated) may also affect results due to physiological heterogeneities in electrophysiology and spatial differences in the response to ischemia. For instance, APD is shorter, steady-state outward potassium current larger, and ischemia-induced APD shortening and outward potassium current increase greater in rabbit isolated ventricular epicardial *versus* endocardial cells (most likely due to differences in *I*_K,ATP_) (Qi et al., 2000). As a result, direct comparison of experimental and computational results is non-trivial and deserves full consideration of potential factors that may affect its outcome.

In our study, comparison with experiments highlights the impact that differences in experimental conditions and preparations have on the time course of ischemic changes. The computational rabbit models proposed here are therefore used as a tool to bridge multiscale experimental data (Carusi et al., 2012, Quinn and Kohl, 2013) to allow exploration of a, as of yet, poorly understood aspect of biology: physiological variability in cardiac electrophysiology and its role in the variable response to pathological situations.

A critical aspect of models in physiology as representational tools is the question of what constitutes a ‘valid’ model. Validation in computational physiology is a term under investigation itself. We have previously suggested that validation in computational physiology is an iterative process that needs to consider a model-simulation-experiment system, with the main aim of increasing our understanding of the physiological system, in this case the heart (Carusi et al., 2012, Noble, 2011, Quinn and Kohl, 2013). Others propose to assess model ‘validity’ purely by statistical means (Johnstone et al., 2016). The idea seems attractive and worth exploring. However, we argue that this is not the only path for validation in computational physiology, and one that would be challenging (to say the least) given the dynamic nature of acute ischemia and limitations of current experimental techniques. Physiological systems (including cardiac cells affected by disease) are not fully known, they cannot be fully characterised experimentally, and are open systems affected by external conditions. Importantly, at present, experimental techniques in cardiac electrophysiology are invasive and provide measurements affected by aggressive procedures. Important for the present study, ionic currents’ conductance can only be experimentally measured by voltage-clamp in isolated cells, a preparation in which ion channel density is adversely impacted (Yue et al., 1996), and not all conductances can be measured in the same preparation at a particular point in time. Thus, the ‘true’ distribution of currents’ conductance is out of reach, and would only be relevant for a particular experiment. In fact, even if we could estimate it, it would quickly become irrelevant as ion channel densities are not parameters, and vary dynamically due to a variety of factors, as an increasing body of research is highlighting.

The population of models methodology (with or without experimental calibration) provides useful tools to explore variability, which need to be adapted for specific investigations, to identify and probe key determinants of physiological variability. In fact, it has been suggested that biological systems are generally tolerant to significant variations of many parameter combinations (Gutenkunst et al., 2007), supported, for instance, by a recent study showing correspondence between gene expression data and a population of mouse ventricular AP models (Weiss et al., 2012).

Our investigations highlight the importance of variability in cardiac electrophysiology, and also highlight the need for consideration of a variety of theoretical approaches, given limitations of experimental measurements. It should be noted that various other effective computational frameworks exist and we hope more will be developed in coming years (Krogh-Madsen et al., 2016, Muszkiewicz et al., 2016), with incorporation also of uncertainty quantification (Mirams et al., 2016, Wallman et al., 2014). Specific model assumptions may also be refined. For example, in previous studies by us and others, the populations of models approach has been implemented using the assumptions that parameters are uniformly distributed and independent. As with any modelling approach in biology, the assumptions need to be evaluated taking into consideration the biological context of the investigations, which includes research questions, experimental datasets, and experimental conditions and protocols.

Alternative methodologies can be used, such as for example a hierarchical Bayesian approach, which involves the search of a parameter space for a defined number of values generating simulated outputs that are close to experimental data with respect to summary statistics. These methodologies can indeed be statistically more powerful than the uniform sampling technique used in our study (Drovandi et al., 2015). However, with Bayesian analysis, translating subjective prior beliefs into a mathematically formulated prior is not straight forward (so can lead to misleading results), and has a strong influence on the posterior distribution (the probability distribution of the parameters, which is conditional on the data) (Gelman, 2008). In fact, when prior knowledge is either vague or non-existent, it has been recommended that a uniform distribution is used (Syversveen, 1998). This is certainly the case in our investigations as currently, due to the experimental constraints discussed above, there is little to no established data available about the distribution of currents’ conductance across large populations. Furthermore, we should note that the assumption of a uniform distribution is not a requirement of the population of models approach, so if warranted, non-uniform distributions (*via* the use of transformations within Latin-Hypercube sampling) can be employed.

In terms of parameter independence, on the other hand, to the best of our knowledge there is no conclusive evidence to suggest that channels’ conductance in the heart are correlated. However, if desired, dependent relationships can be imposed when using population of models methodology to explore the potential contribution of this aspect (for instance, a reciprocal modulation of *I*_K1_ and *I*_Na_ has recently been demonstrated within a macromolecular complex (Milstein et al., 2012)). Alternatively, a thorough study based on Latin-Hypercube sampling can first be conducted to identify correlations, followed by resampling of a smaller space.

Ultimately, it is important to recognise that this is an exciting area of research at the intersection of experimental and computational physiology, and currently there is insufficient experimental or computational evidence to support the exclusive use of any one method to study variability. This is an area under investigation and the different approaches being proposed by various groups each have advantages and limitations for specific research contexts. They may in fact all be important for investigating variability. It seems unlikely that one method will ultimately prove to be superior to the rest for all applications, especially given the limitations of current experimental datasets and techniques. Therefore, it is important to embrace and explore the potential contribution of the diversity of methods that is being suggested to investigate variability in cardiac electrophysiology, which considers diverse biological and mathematical viewpoints.

## 5 Conclusions

In this paper we have briefly reviewed current progress in rabbit-specific computational modelling of ventricular cell electrophysiology and its use for studying the effects of cellular heterogeneity on electrical activity. This was followed by presentation of a computational framework using a population of models approach to study the effects of physiological cellular variability in the response to acute ischemia. Using two rabbit-specific computational ventricular AP models that reproduce experimentally-observed variability of APD, we demonstrated enhanced variability in cellular electrophysiology during acute ischemia, which may have important implications for arrhythmogenesis. This represents a method that may be extended to other disease states, as well as in multi-cellular simulations to include a contribution of cellular variability to tissue heterogeneity, helping define the role that physiological variability plays in determining arrhythmic risk.

## Figures and Tables

**Table 1:** Values of ischemic parameters for simulation of varying degrees of ischemic severity (for simplicity referred to by time, although time course is dependent on experimental conditions and differs across regions of the rabbit ventricles).

Time of ischemia (min)	0	2	4	6	8	10
*f*_K(ATP)_ (%)	0.00	0.16	0.32	0.48	0.64	0.80
*f*_inhib_ (%)	0	5	10	15	20	25
*f*_NaK_ (%)	0	6	12	18	24	30
[K^+^]_o_ (mM)	5.4	6.7	8.0	9.4	10.7	12.0
	5.4	7.3	9.2	11.2	13.1	15.0
	5.4	7.7	10.0	12.4	14.7	17.0

**Table 2:** Currents’ conductance in control conditions.

Model population	*g*_to_	*g*_Ca,L_	*g*_Kr_	*g*_Ks_	*g*_K1_	*g*_NaK_
Shannon	+30%	+0%	+0%	−15%	−30%	+15%
Mahajan	+0%	+75%	+30%	+75%	+0%	−30%

Values represent percentage change from original model value.

## Editors’ note

Please see also related communications in this issue by Arevalo et al. (2016) and Rog-Zielinska et al. (2016).
